# Inflammatory Cytokine Genetics and Coronary Artery Disease: Pathogenetic and Protective Analysis of IL-18 (−607 C/A, −137 G/C) and IL-8 (+781 C/T) Gene Variations

**DOI:** 10.3390/cimb48060589

**Published:** 2026-06-02

**Authors:** Arzu Ay, Nevra Alkanli, Gokay Taylan, Esra Ergin

**Affiliations:** 1 Department of Biophysics, Faculty of Medicine, Trakya University, Edirne 22030, Türkiye; esraergin1@hotmail.com; 2 Department of Biophysics, Faculty of Medicine, Haliç University, Istanbul 34060, Türkiye; nevraalkanli@halic.edu.tr; 3 Department of Cardiology, Faculty of Medicine, Trakya University, Edirne 22030, Türkiye; gokaytaylan@trakya.edu.tr

**Keywords:** coronary artery disease, interleukin 18, interleukin 8, variation, genetic risk, protective effect

## Abstract

Chronic inflammation mediated by cytokines is central to the pathogenesis of coronary artery disease (CAD). This exploratory study aimed to investigate potential associations between functional gene variations of the cytokines interleukin 18 (IL-18) and interleukin 8 (IL-8) and the CAD susceptibility within a specific regional cohort, while accounting for common clinical comorbidities. Genotype distributions of IL-18 (−607 C/A, −137 G/C) and IL-8 (+781 C/T) were analyzed in a cohort of 102 patients with angiographically confirmed CAD and 102 healthy controls. Genotyping was performed using PCR, allele-specific PCR, and RFLP techniques. Multivariate logistic regression was utilized to assess potential independent associations, adjusting for age and traditional clinical risk factors. In this specific cohort, after adjusting for age, hypertension, diabetes, cholesterol, family history, and smoking, the IL-18 (−137 G/C) CC genotype was observed more frequently in CAD patients (adjusted odds ratio [AOR] = 6.15, 95% CI = 2.10–18.05, *p* = 0.001). An exploratory analysis of genotype combinations suggested that the IL-18 (−607/−137) CA-CC profile may be linked to an increased risk (AOR = 3.65, *p* = 0.028), while tentative protective trends were noted for certain IL-18/IL-8 combinations. Notably, a significant deviation from a Hardy–Weinberg equilibrium (HWE) was observed in the control group for the IL-18 loci, which represents a substantial methodological limitation that may influence these risk estimates. Our preliminary findings suggest that specific IL-18 and IL-8 variations may contribute to CAD susceptibility independently of traditional risk factors in the studied population. However, given the modest sample size and the observed HWE deviation, these associations should be regarded as suggestive rather than definitive. While these genetic variations underscore the importance of cytokine pathways in cardiovascular research, they do not currently support clinical implementation for risk stratification. Large-scale, multi-center prospective studies are necessary to validate these preliminary signals and evaluate their long-term scientific utility.

## 1. Introduction

Coronary artery disease (CAD) remains the leading cause of death and disability worldwide, despite various technological advances in modern medicine [1,2]. The pathogenesis of atherosclerosis is considered a chronic inflammatory process during all stages, from its onset to plaque rupture [3,4]. Endothelial dysfunction following lipid accumulation in the vessel wall triggers the migration of immune system cells to the lesion site and the release of pro-inflammatory cytokines from these cells, which exacerbate vascular damage [2,4]. Genome-wide association studies (GWASs) conducted in recent years have revealed that the severity of this inflammatory response and individuals’ susceptibility to CAD are largely determined by genetic variations [1,2].

Interleukin 18 (IL-18) is a cytokine initially defined as an interferon γ (IFN-γ)-inducing factor that plays a central role in innate and adaptive immune responses [3,5]. IL-18 is highly expressed within atherosclerotic plaques and facilitates plaque rupture (unstable plaques) by increasing the production of metalloproteinases [1,3]. Current meta-analyses confirm that variations in the promoter region of IL-18 exert direct control over protein expression [1]. Specifically, the −607 C/A (rs1946518) and −137 G/C (rs187238) variants modulate IL-18 levels by altering the binding affinity of transcription factors [1,5]. Studies report that the IL-18 levels are an independent predictor of mortality in ischemic heart disease [3].

Interleukin 8 (IL-8)—another chemokine that plays a key role in atherosclerosis—is the primary molecule that enables the chemotaxis of leukocytes from the vascular lumen to the intima layer [2,4]. The IL-8 +781 C/T (rs2227306) variation might determine the rate of lesion progression by affecting the production capacity of IL-8 during vascular inflammation [4]. Although IL-8 variants are reportedly strongly associated with CAD susceptibility, particularly in Asian populations, this effect varies across different ethnicities and in the presence of comorbidities [2,4].

In clinical practice, the role of traditional risk factors, such as diabetes, hypertension, and smoking, in the development of CAD is well established. However, the question of why the disease progresses differently in individuals with similar clinical risk profiles has necessitated the examination of gene–environment interactions and haplotype structures [2,5]. The analysis of single-nucleotide polymorphisms (SNPs) can sometimes be insufficient; blocks of genetic variation inherited together (haplotypes) provide a more comprehensive perspective for understanding the molecular basis of disease [2].

The aim of this study was to investigate the frequencies of IL-18 (−607 C/A, −137 G/C) and IL-8 (+781 C/T) gene variations in individuals diagnosed with CAD within a Thracian cohort. Furthermore, the study sought to explore the potential associations of these genetic variants with disease risk—individually and at the haplotype/multi-locus levels—while evaluating their possible modulatory effects on the clinical profiles (e.g., diabetes, hypertension, and family history) of the patients.

## 2. Materials and Methods

### 2.1. Study Design and Ethical Approval

This retrospective case–control study enrolled 102 patients with CAD and 102 healthy controls. The research protocol was reviewed and accepted by the Trakya University Faculty of Medicine Non-Interventional Scientific Research Ethics Committee and declared ethically acceptable (approval No.: TÜTF-GOBAEK 2023/119). During the research process, the current standards of the Declaration of Helsinki, which covers ethical principles for medical research involving humans, were strictly adhered to. All the participants included in the study were informed verbally and in writing about the purpose, scope, and genetic analysis processes of the research; then, signed informed consent forms were obtained from the participants. Based on the study by Mitrokhin and colleagues, the effect size was set at 0.25 and the α value at 0.05 for the power analysis, giving a power value (1-β) of 0.90 [6]. Accordingly, the sample size was calculated at a minimum of 204. As such, 102 individuals were included for the CAD and control groups each.

### 2.2. Characterization of CAD and Control Groups

The 102 participants in the CAD group were followed up by the cardiology clinic and diagnosed with CAD, which was confirmed by angiography. In addition, 102 healthy individuals without CAD were included. When creating the groups, care was taken to ensure that basic demographic characteristics, such as age, were similar to ensure statistical comparability. The CAD group consisted of individuals who visited the cardiology outpatient clinic and ward, had at least a 50% or greater stenosis detected in one main coronary artery as verified by coronary angiography, and had a confirmed diagnosis of CAD. The mean age of the CAD group was 66.91 ± 10.67 years. The control group consisted of individuals with similar age characteristics to the CAD group who had no history of CAD, had normal coronary arteries by angiographic or clinical evaluations, and were healthy volunteers. The control group comprised two subsets to ensure a representative healthy population. The first subset included symptomatic individuals who underwent coronary angiography due to suspected CAD, but had angiographically normal coronary arteries (i.e., no detectable plaques or stenosis). The second subset consisted of asymptomatic, clinically healthy volunteers with no history of cardiac symptoms or abnormal findings from non-invasive clinical evaluations (e.g., physical examination or ECG). This dual approach was adopted to balance the ethical constraints of invasive procedures with the need for a verified healthy baseline group. The mean age of the control group was 66.35 ± 7.89 years. The inclusion criteria for the CAD group were an age of 18 years or older and angiographically documented CAD. For the control group, the criteria were being aged 18 years or older, having no diagnosis of CAD, and having normal results from cardiac tests (e.g., effort test, ECG, or angiography). Individuals with conditions that could affect the genetic or inflammatory outcomes of the study were excluded. Individuals with an active infection or chronic inflammatory disease or who were diagnosed with any malignancies, severe liver or kidney failure, an autoimmune disease, or hematological disorders were excluded from the study. The participants’ demographic information and clinical histories were collected from hospital records.

### 2.3. Genetic Analysis and Genotyping

Peripheral venous blood samples were collected from all the participants and genomic DNA isolation was performed using standard methods. The isolated DNA samples were analyzed with polymerase chain reaction (PCR), allele-specific PCR, and RFLP techniques to determine the presence of IL-18 (−607 C/A, −137 G/C) and IL-8 (+781 C/T) gene variations. Genotyping was performed using two different PCR-based techniques according to the structural requirements of the targeted loci. For IL-18 (rs1946518 and rs187238), allele-specific PCR was used. Two specific forward primers were designed for each variant to distinguish between wildtype and mutant alleles, along with a common reverse primer. The presence of a specific PCR product indicated the corresponding genotype. No restriction enzyme digestion was required for these loci. For IL-8 (rs2227306), PCR and RFLP methods were used. After the initial amplification of the target sequence, the PCR products were subjected to enzymatic digestion using the EcoRI restriction enzyme (Thermo Scientific, Waltham, MA, USA) at 37 °C for 16 h. The resulting fragments were visualized on a 2% agarose gel stained with ethidium bromide. All PCRs contained 50 ng of isolated DNA, appropriate forward and reverse primer sets, 1x PCR buffer, 3 mM MgCl_2_, and 1.25 units of Taq DNA polymerase. A 40-cycle amplification protocol was applied with specific primer sequences for the IL-18 (−607 C/A) and IL-18 (−137 G/C) regions. An annealing temperature of 50 °C was selected for the IL-18 (−607 C/A) region and 54 °C for the IL-18 (−137 G/C) region. The allele-specific PCR products were visualized with 2% agarose gel electrophoresis. A 35-cycle PCR protocol (annealing temperature of 61 °C) was used for the IL-8 (+781 C/T) region and the final extension step was 72 °C for 20 min. The amplified DNA regions were cut with the appropriate restriction enzyme to distinguish genetic variation. The RFLP mixture was prepared with 1x buffer Tango, PCR products, dH_2_O, and 5 units of restriction enzyme. The band profiles formed after enzyme cutting were analyzed using 2.5% agarose gel electrophoresis. For IL-18 (−607 C/A) genotyping, the CC, CA, and AA genotypes were identified based on the reactions from the Forward 1 and Forward 2 primers for the 196 bp products. For IL-18 (−137 G/C) genotyping, the GG, GC, and CC genotypes were identified based on the 261 bp product profiles. For IL-18 (−607 C/A) gene variation, the CC genotype was observed as 196 bp products (Forward 1 primer), the CA genotype as 196 bp products (Forward 1 and Forward 2 primers), and the AA genotype as 196 bp products (Forward 2 primer) (Figure S1). For IL-18 (−137 G/C) gene variation, the GG genotype was identified as 261 bp products (Forward 1 primer), the GC genotype as 261 bp products (Forward 1 and Forward 2 primers), and the CC genotype as 261 bp products (Forward 2 primer) (Figure S2). For IL-8 (+781 C/T) gene variation, the TT genotype was represented by a single 203 bp band. The CT genotype showed two 203 and 184 bp bands (the 19 bp band was not observable). The CC genotype produced a 184 bp band due to complete enzyme cleavage (Figure S3). Detailed information regarding primer sequences, reaction conditions, and specific genotyping protocols for allele-specific PCR and RFLP is provided in Supplemental Files S1 and S2, where the methodologies are categorized by technique to ensure technical clarity.

### 2.4. Statistical Analysis

Statistical analyses were performed using IBM SPSS Statistics, Version 26.0 (IBM Corp., Armonk, NY, USA). Continuous variables, such as age, were expressed as the mean ± standard deviation (SD) and compared using an independent-sample *t*-test. Categorical variables—including clinical characteristics (hypertension, diabetes, hypercholesterolemia, smoking status, and family history) and genotype distributions—were presented as frequencies and percentages. Differences between the CAD and control groups were evaluated using chi-squared or Fisher’s exact tests where appropriate. Genotype distributions were tested for deviation from the Hardy–Weinberg equilibrium (HWE) in both groups. For quality control, 10% of the samples were re-analyzed with 100% reproducibility.

To determine the risk associated with specific genotypes, haplotypes/multi-locus genotype combinations, and clinical factors, the odds ratios (ORs) and 95% confidence intervals (CIs) were calculated using logistic regression models. Given the baseline imbalances in cardiovascular risk factors between the groups, a multivariate logistic regression analysis (enter method) was performed. This model included age and all significant clinical parameters (hypertension, diabetes, cholesterol, family history, and smoking) as covariates to calculate the adjusted ORs (AORs), thereby isolating the independent effect of the IL-18 and IL-8 variations.

A two-tailed *p*-value of less than 0.05 was considered statistically significant for all analyses. However, to address the burden of multiple testing in the primary association analyses of the three SNPs, a Bonferroni-corrected threshold of *p* < 0.0167 (0.05/3) was applied. Conversely, subgroup analyses based on clinical parameters and multi-locus genotype combinations were treated as exploratory; for these specific analyses, nominal *p*-values are reported to maintain transparency and generate hypotheses, as highlighted in the respective tables.

## 3. Results

### 3.1. Comparison of Demographic and Clinical Parameters

The CAD group (mean age: 66.91 ± 10.67 years) and the control group (mean age: 66.35 ± 7.89 years) were well-matched regarding age (*p* = 0.673). However, significant disparities were observed between the two groups regarding clinical risk factors. The prevalence of hypertension (56.9%), diabetes (58.8%), and hypercholesterolemia (53.9%) was significantly higher in the CAD group compared with the control cohort (*p* < 0.001). Similarly, a family history of CAD (55.9%) and smoking (61.8%) were notably more frequent in patients with CAD (*p* < 0.001). No statistically significant differences were observed between the groups regarding alcohol consumption (*p* = 0.061) (Supplemental File S3).

### 3.2. Independent Predictors of CAD (Multivariate Analysis)

To evaluate the independent contributions of clinical and genetic variables, a multivariate logistic regression analysis was conducted, adjusting for age and all statistically significant clinical covariates. Table 1 shows that traditional risk factors, including hypertension (adjusted OR [AOR] = 4.12, 95% CI = 2.05–8.28, *p* < 0.001), diabetes mellitus (AOR = 3.85, 95% CI = 1.92–7.72, *p* < 0.001), and hypercholesterolemia (AOR = 4.68, 95% CI = 2.24–9.75, *p* < 0.001), were identified as significant independent predictors of the CAD risk in this cohort. Furthermore, while initial univariate findings provide a preliminary overview, a family history of CAD (AOR = 3.22, 95% CI = 1.64–6.33, *p* = 0.001) and smoking (AOR = 2.28, 95% CI = 1.18–4.42, *p* = 0.014) maintained their independent statistical significance within the adjusted model (Supplemental File S3, Table 1). In addition to initial univariate findings, the exploratory multivariate analysis indicated that the IL-18 (−137 G/C) CC genotype was associated with an increased CAD risk (AOR = 6.15, 95% CI = 2.10–18.05, *p* = 0.001) (Table 1 and Table 2); however, this estimate should be viewed within the context of the identified HWE deviation.

### 3.3. Genotype Distributions and CAD Risk

The genotype distributions of the IL-18 and IL-8 variations and their respective associations with the CAD risk are summarized in Table 3. A univariate analysis revealed that the IL-18 (−607 C/A) AA homozygous genotype was more prevalent in the CAD group compared with the controls (17.6% vs. 5.9%), suggesting a 3.42-fold increase in risk (OR = 3.429, 95% CI = 1.301–9.038, *p* = 0.013). Conversely, the heterozygous CA genotype was more frequent in the control group (69.6% vs. 53.0%), appearing to be associated with a reduced risk (OR = 0.491, 95% CI = 0.277–0.872, *p* = 0.015).

For the IL-18 (−137 G/C) variation, the CC genotype was significantly more frequent in patients with CAD (17.6%) than in the control group (4.9%), indicating an increased risk (OR = 4.157, 95% CI = 1.480–11.679, *p* = 0.007). Regarding the IL-8 (+781 C/T) variant, the CC genotype was identified as a potential risk factor (OR = 2.443, 95% CI = 1.364–4.378, *p* = 0.003), whereas the TT genotype showed a substantial protective profile (OR = 0.073, 95% CI = 0.017–0.319, *p* < 0.001) in this cohort (Table 3).

While these initial univariate findings provide a preliminary overview, they do not account for clinical confounders and should be considered exploratory, particularly given the identified HWE deviations (Table 2 and Table 3). To assess the independent associations of these variants, multivariate logistic regression was conducted (Table 1).

Adjusted Analysis Results:

IL-18 (−607 C/A): The AA genotype remained an independent factor associated with risk (AOR = 3.85, 95% CI = 1.10–13.40, *p* = 0.035). Notably, the CA genotype, which exhibited a protective trend in the univariate model, emerged as an independent risk factor after clinical adjustment (AOR = 1.72, 95% CI = 1.01–2.95, *p* = 0.046). This shift suggests a possible suppressor effect or negative confounding by clinical comorbidities in the raw data (Table 1).

IL-18 (−137 G/C): The CC genotype was confirmed as a significant independent predictor, with the risk estimate increasing to 6.15-fold (AOR = 6.15, 95% CI = 2.10–18.05, *p* = 0.001). However, this elevated estimate should be interpreted with caution due to the modest sample size and HWE imbalance. The GC genotype also reached independent significance (AOR = 2.10, 95% CI = 1.15–3.82, *p* = 0.015) (Table 1).

IL-8 (+781 C/T): The risk associated with the CC genotype remained significant (AOR = 2.55, 95% CI = 1.30–5.02, *p* = 0.006), and the CT genotype was similarly identified as an independent risk factor (AOR = 1.88, 95% CI = 1.20–3.15, *p* = 0.007) (Table 1).

These comparative results emphasize the importance of multivariate modeling in uncovering the independent associations between cytokine variations and CAD, although the exploratory nature of these findings remains a primary consideration.

### 3.4. Hardy–Weinberg Equilibrium

To assess the representativeness of the genotype distributions and screen for potential population stratification or genotyping artifacts, a Hardy–Weinberg equilibrium (HWE) analysis was conducted for each locus. For the IL-18 (−137 G/C) and IL-18 (−607 C/A) variants, the genotype distributions were consistent with equilibrium in the CAD group (*p* > 0.05). However, statistically significant deviations from the HWE were observed in the control group for these loci (*p* = 0.003 and *p* = 0.0006, respectively). These departures in the control cohort may be attributed to inherent population characteristics of the Thrace region—such as the Wahlund effect resulting from intense migration and regional endogamy—as well as the modest sample size and specific selection criteria. We acknowledge that these deviations represent a significant limitation, potentially affecting the internal validity and generalizability of the IL-18 associations.

In contrast, the IL-8 (+781 C/T) variation was consistent with the HWE in the control group (*p* = 0.811), while a significant deviation was detected in the patient group (*p* < 0.001). Such deviations in the case arm are often anticipated in genetic association studies when a locus is strongly linked to the disease phenotype, as reflected by the independent risk associated with this variant in our multivariate model (AOR = 2.55, 95% CI = 1.30–5.02, *p* = 0.006) (Table 1).

To ensure technical reliability, all genotype calls were validated by re-genotyping 10% of the samples, which yielded 100% concordance and a 100% call rate for all variants. While these quality control measures suggest that the HWE findings are unlikely to have stemmed from technical genotyping errors, the observed imbalances in the IL-18 loci necessitate a cautious interpretation of the genetic risk estimates, as they may reflect complex underlying genetic architectures or cryptic relatedness within the studied population.

### 3.5. Haplotype and Multi-Locus Genotype Combination Analysis

To identify potential synergistic effects between the investigated loci, haplotype and multi-locus genotype combination analyses were performed. Given the increased number of variables relative to the sample size, these findings are framed strictly as exploratory and are intended to provide nominal, hypothesis-generating signals rather than robust independent predictors (Table 4).

The univariate analysis of IL-18 (−607 C/A) and IL-18 (−137 G/C) combinations suggested that the CA-CC haplotype was associated with an increased CAD risk (OR = 4.22, 95% CI = 1.351–13.209, *p* = 0.013). Conversely, the CA-GC haplotype was more prevalent in the control group (46.1%) than in the CAD group (26.5%), suggesting a potential protective trend (OR = 0.421, 95% CI = 0.234–0.758, *p* = 0.004). Regarding IL-18 (−607 C/A) and IL-8 (+781 C/T) combinations, the CC-CC and AA-CC profiles showed nominal associations with an increased CAD risk (OR = 3.086, 95% CI = 1.068–8.918, *p* = 0.037 and OR = 3.267, (1.017–10.496), *p* = 0.046, respectively). In contrast, the CC-TT combination was observed in 9.8% of the controls, but only 2.0% of the patients (OR = 0.184, 95% CI = 0.039–0.862, *p* = 0.032), while the CA-TT combination also appeared significantly less frequently in the CAD group (OR = 0.150, 95% CI = 0.033–0.689, *p* = 0.015). Notably, the IL-8 TT variant appeared to maintain a consistent protective trend, even when co-occurring with various IL-18 alleles (Table 4).

Furthermore, in the IL-18 (−137 G/C) and IL-8 (+781 C/T) multi-locus analysis, the GG-CC combination was linked to a 2.27-fold increase in risk (OR = 2.275, 95% CI = 1.039–4.981, *p* = 0.040). Conversely, the GC-TT combination was more frequent in the controls (11.8%) than in the patients (2.0%), appearing to reduce the CAD susceptibility (OR = 0.150, 95% CI = 0.033–0.689, *p* = 0.015). These exploratory observations suggest that the IL-8 (+781 C/T) CC genotype, in potential synergy with IL-18 variants, may be associated with CAD, while the TT genotype—alone and in specific combinations (CA-TT, GC-TT)—appears to represent a less susceptible genetic profile (Table 4).

Building upon these preliminary findings (Table 4), multivariate logistic regression was conducted to evaluate the independent associations of these combinations after adjusting for clinical factors (Table 1). Due to the limited statistical power in these subgroups, these adjusted results should be interpreted with extreme caution.

Exploratory independent risk associations: The IL-18 (−607/−137) CA-CC combination was confirmed as an independent risk factor (adjusted OR [AOR] = 3.65, 95% CI = 1.12–11.85, *p* = 0.028) (Table 1).

Exploratory independent protective associations: The CA-GC combination of IL-18 (−607/−137) remained associated with a reduction in risk (AOR = 0.48, 95% CI = 0.26–0.89, *p* = 0.021). Additionally, the IL-18 (−607)/IL-8 (+781) CA-TT combination was identified as an independent protective marker (AOR = 0.18, 95% CI = 0.04–0.82, *p* = 0.027) (Table 1).

Non-significant associations (clinical confounding): While the CC-CC and AA-CC combinations of IL-18 (−607)/IL-8 (+781) showed nominal significance in the univariate analysis, they did not reach statistical significance in the multivariate model (AOR = 2.65, 95% CI = 0.92–7.64, *p* = 0.071 and AOR = 2.88, 95% CI = 0.90–9.25, *p* = 0.075, respectively). This loss of significance suggests that these observed effects were likely influenced by clinical covariates rather than representing independent genetic contributions (Table 1).

In summary, while these results point toward specific synergistic trends, particularly regarding the CA-CC risk profile and the protective influence of the IL-8 TT allele, they represent exploratory signals that strictly require validation in much larger, independent cohorts to confirm their stability.

## 4. Discussion

Cardiovascular diseases remain the leading cause of global mortality, with coronary artery disease (CAD) representing a complex multifactorial inflammatory process [7,8]. While the interplay between environmental triggers and genetic predisposition is well-recognized, the specific molecular architecture underlying disease susceptibility often exhibits significant inter-ethnic variability [9,10,11,12,13,14]. Therefore, characterizing genetic variants within specific regional cohorts is a valuable step toward understanding the broader landscape of vascular inflammation [8,12].

Our study explored potential associations between functional variations in the pro-inflammatory cytokine genes *IL*-18 and *IL*-8 and CAD within a Thracian cohort. *IL*-18 is a pleiotropic cytokine primarily known for inducing IFN-γ and contributing to the destabilization of atherosclerotic plaques [1,3,15]. Beyond its established role in vascular pathologies, current evidence highlights IL-18 as a critical mediator in infectious, metabolic, and chronic inflammatory conditions [16,17,18]. In the present study, the *IL*-18 (−137 G/C) CC genotype was observed as an independent factor associated with CAD risk (AOR = 6.15, 95% CI = 2.10–18.05). Although this direction of association aligns with findings in certain Asian populations [5], the magnitude of the effect observed here is substantially higher than those typically reported in larger Caucasian datasets [1,2]. We explicitly acknowledge that this high odds ratio must be interpreted with extreme caution. It is highly probable that this value was inflated by our modest sample size and the unresolved HWE deviation in the control group. Consequently, this finding should be viewed as a preliminary signal requiring verification in larger, higher-powered cohorts.

A critical observation in our multivariate analysis was the reversal of the effect direction for the *IL*-18 (−607 C/A) CA genotype. Initially exhibiting a protective trend in the univariate analysis (OR = 0.491), it emerged as a potential risk factor after adjusting for clinical covariates (AOR = 1.72, *p* = 0.046). This statistical shift requires careful justification. Such reversals often indicate a ‘suppressor effect’ or ‘negative confounding,’ where the independent contribution of a genetic variant is masked in unadjusted data by predominant clinical risk factors—such as age, hypertension, and smoking—that may be disproportionately distributed between carriers. The multivariate model revealed a previously suppressed signal, suggesting that the CA variant may independently contribute to CAD susceptibility when its interaction with age and hypertension is statistically partitioned. The transition from a protective to a risk-conveying profile across models acknowledges the complexity of these interactions and suggests that the adjusted results should be approached with caution. While this underscores the importance of multifactorial modeling, the instability of this association across models limits the confidence with which these results can be generalized. We present this as an exploratory finding that necessitates further validation to confirm the stability of the independent risk.

Regarding IL-8 (CXCL8), this chemokine is recognized for its role in facilitating leukocyte migration into the vascular wall, which may influence the intensity of the local inflammatory response [2,4,19]. Our data suggest a potential protective profile for the (+781 C/T) TT genotype, particularly within certain combinations. However, we must emphasize the preliminary and tentative nature of these findings. Furthermore, all multi-locus and subgroup analyses (e.g., associations in smokers or familial cases) are framed strictly as exploratory. In a study of this size, these subgroup findings cannot be regarded as robust independent predictors. Instead, they serve as nominal, hypothesis-generating observations that provide a scientific baseline for future longitudinal research. We explicitly acknowledge that these *p* < 0.05 associations are secondary findings and should be viewed as areas for future investigation rather than established risk markers. These associations do not, at this stage, support definitive clinical risk stratification.

Despite the identified associations, substantial limitations warrant a transparent interpretation of the data. Most notably, the significant deviation from the Hardy–Weinberg equilibrium (HWE) in the control group for both IL-18 loci is a major unresolved concern. While we hypothesize that this may reflect the Wahlund effect—a reduction in heterozygosity caused by the unique demographic history, intense migration, and regional endogamy of the Thrace region [20,21,22]—we concede that this imbalance remains a primary constraint. Such a departure from the HWE indicates that the control group may not fully represent the general population, which could potentially bias the risk estimates and limit the internal validity of the genetic association.

Furthermore, the modest sample size restricted the statistical power of the study, particularly for rare haplotype and multi-locus analyses. Consequently, our data do not support the identification of robust biomarkers or the implementation of personalized screening tools. Additionally, the lack of circulating cytokine data prevents a direct functional correlation between the identified genotypes and protein expression levels. Without such validation, the mechanistic link remains suggestive rather than confirmed.

In summary, while our findings suggest that functional variations in the IL-18 and IL-8 genes may be associated with CAD susceptibility in this cohort, these results represent a preliminary molecular framework rather than conclusive clinical evidence. The observed HWE deviation and the single-center design necessitate significant caution in interpretation. Future large-scale, multi-center prospective studies are strictly required to validate these exploratory signals and to evaluate their eventual scientific utility in targeted prevention strategies.

## 5. Conclusions

In conclusion, this exploratory study suggests a potential association between functional variations in the IL-18 and IL-8 genes and CAD susceptibility within the studied Thracian cohort. While multivariate modeling points toward these genetic trends persisting alongside traditional risk factors, these findings must be viewed strictly as preliminary and hypothesis-generating rather than definitive evidence of genetic risk.

Specifically, the IL-18 (−137 G/C) CC genotype was observed more frequently in the CAD group. However, the magnitude of the observed adjusted OR (6.15) must be interpreted with extreme caution, as it may have been substantially inflated by the study’s modest sample size and the significant deviation from the Hardy–Weinberg equilibrium (HWE) in the control group. Similarly, the trends observed for the IL-18 (−607 C/A) AA genotype and various multi-locus combinations may serve only as potential candidates for further investigation into pro-inflammatory pathways, rather than established risk predictors.

Regarding the IL-8 (+781 C/T) TT genotype, our data show a trend toward a protective association. Nevertheless, the stability and biological relevance of this effect remain unverified and require confirmation through large-scale, multi-center prospective studies.

We explicitly acknowledge that the small cohort size and HWE deviations represent substantial constraints that limit the generalizability of our findings. Consequently, our results do not support the use of these SNPs as clinical biomarkers or personalized screening tools at this stage. Instead, they suggest a biological basis that warrants further longitudinal studies. Ultimately, while these data highlight the IL-18 and IL-8 pathways as areas of academic interest, large-scale functional and multi-ethnic studies are strictly required to determine if these markers hold any eventual value for risk stratification or targeted anti-inflammatory strategies.

## Figures and Tables

**Table 1:** Multivariate logistic regression analysis of all genetic variations, multi-locus genotype combinations, and clinical predictors.

***Variable***	***Univariate OR* (95% *CI*)**	***Adjusted OR* (95% *CI*)**	***p-Value* (*Adjusted*)**
***Clinical Covariates***			
Age	1.01 (0.97–1.04)	1.02 (0.98–1.06)	0.695 ^a^
Hypertension (+)	7.646 (3.898–14.996)	4.12 (2.05–8.28)	<0.001 *
Diabetes mellitus (+)	7.143 (3.717–13.726)	3.85 (1.92–7.72)	<0.001 *
Cholesterol (+)	8.012 (3.978–16.136)	4.68 (2.24–9.75)	<0.001 *
Familial history of CAD (+)	6.333 (3.303–12.145)	3.22 (1.64–6.33)	0.001 *
Alcohol (+)	1.818 (0.972–3.401)	1.45 (0.74–2.85)	0.281
Smoking (+)	3.700 (2.070–6.614)	2.28 (1.18–4.42)	0.014 *
***Genetic Variations***	***Univariate OR*** **(95% *CI*)**	***Adjusted OR*** **(95% *CI*)**	***p-Value*** **(*Adjusted*)**
IL-18 (−607 C/A) AA	3.429 (1.301–9.038)	3.85 (1.10–13.40)	0.035 *
IL-18 (−607 C/A) CA	0.491 (0.277–0.872)	1.72 (1.01–2.95)	0.046 *
IL-18 (−137 G/C) CC	4.157 (1.480–11.679)	6.15 (2.10–18.05)	0.001 *
IL-18 (−137 G/C) GC	0.594 (0.340–1.038)	2.10 (1.15–3.82)	0.015 *
IL-8 (+781 C/T) CC	2.443 (1.364–4.378)	2.55 (1.30–5.02)	0.006 *
IL-8 (+781 C/T) CT	0.962 (0.555–1.665)	1.88 (1.20–3.15)	0.007 *
***Haplotypes Multi-locus Genotype Combinations***	***Univariate OR*** **(95% *CI*)**	***Adjusted OR*** **(95% *CI*)**	***p-Value*** **(*Adjusted*)**
IL-18 (-607/-137) CA-CC	4.224 (1.351–13.209)	3.65 (1.12–11.85)	0.028 *
IL-18 (-607/-137) CA-GC	0.421 (0.234–0.758)	0.48 (0.26–0.89)	0.021 *
IL-18 (-607)/IL-8 CC-CC	3.086 (1.068–8.918)	2.65 (0.92–7.64)	0.071
IL-18 (-607)/IL-8 AA-CC	3.267 (1.017–10.496)	2.88 (0.90–9.25)	0.075
IL-18 (-607)/IL-8 CA-TT	0.150 (0.033–0.689)	0.18 (0.04–0.82)	0.027 *

OR: Odds ratio. CI: Confidence interval. CAD: Coronary artery disease. * Statistical significance (*p* < 0.05), given in bold italic font. Notes: All variables (clinical and genetic) were entered into a single multivariate logistic regression model to determine independent risk. Model adjusted for age, hypertension, diabetes mellitus, cholesterol, familial history of CAD, and smoking status.

**Table 2:** Genotype distributions of IL-18 and IL-8 gene variations according to comorbidities.

***Genotype Distributions***		***CAD Group* (*n* = 102),** ***Diabetes* (+/−)**		***OR*, 95% *CI***	***p-Value***
***IL*-18 ** **(−607 *C*/*A*)**	CC	18 (17.7%)	17 (16.6%)	1.071, (0.517–2.219)	0.853 ^a^
	CA	33 (32.4%)	20 (19.6%)	1.961, (1.033–3.723)	**0.040 *^a^*** *
	AA	9 (8.8%)	5 (4.9%)	1.877, (0.607–5.810)	0.274 ^a^
***IL*-18 ** **(−137 *G*/*C*)**	CC	9 (8.8%)	6 (5.9%)	1.548, (0.530–4.522)	0.424 ^a^
	GC	36 (35.2%)	18 (17.7%)	2.546, (1.327–4.882)	**0.005 *^a^*** *
	GG	15 (14.7%)	18 (17.7%)	0.805, (0.381–1.701)	0.569 ^a^
***IL*-8 ** **(+781 *C*/*T*)**	CC	26 (25.5%)	23 (22.6%)	1.175, (0.618–2.236)	0.623 ^a^
	CT	34 (33.3%)	19 (18.6%)	2.184, (1.444–4.169)	**0.018 *^a^*** *
	TT	-	-	-	-
***Genotype Distributions***		***CAD Group*** **(*n* = 102),** ***Hypertension*** **(+/−)**		***OR*, ** ** 95% *CI***	***p-Value***
***IL*-18 ** **(−607 *C*/*A*)**	CC	17 (16.6%)	14 (13.7%)	1.257, (0.584–2.709)	0.559 ^a^
	CA	32 (31.5%)	25 (24.5%)	1.408, (0.761–2.605)	0.276 ^a^
	AA	9 (8.8%)	5 (4.9%)	1.877, (0.607–5.810)	0.274 ^a^
***IL*-18 ** **(−137 *G*/*C*)**	CC	9 (8.8%)	6 (5.9%)	1.548, (0.530–4.522)	0.424 ^a^
	GC	31 (30.5%)	22 (21.5%)	1.588, (0.843–2.990)	0.152 ^a^
	GG	18 (17.6%)	16 (15.7%)	1.152, (0.551–2.408)	0.707 ^a^
***IL*-8 ** **(+781 *C*/*T*)**	CC	23 (22.6%)	26 (25.5%)	0.851, (0447–1.619)	0.623 ^a^
	CT	35 (34.3%)	18 (17.6%)	2.438, (1.269–4.683)	**0.008 *^a^*** *
	TT	-	-	-	-
***Genotype Distributions***		***CAD Group*** **(*n* = 102),** ***Cholesterol*** **(+/−)**		***OR*, ** ** 95% *CI***	***p-Value***
***IL*-18 ** **(−607 *C*/*A*)**	CC	16 (15.7%)	15 (14.7%)	1.079, (0.502–2.319)	0.845 ^a^
	CA	30 (29.4%)	27 (26.5%)	1.157, (0.627–2.135)	0.640 ^a^
	AA	9 (8.8%)	5 (4.9%)	1.877, (0.607–5.810)	0.274 ^a^
***IL*-18 ** **(−137 *G*/*C*)**	CC	8 (7.9%)	7 (6.9%)	1.155, (0.403–3.313)	0.789 ^a^
	GC	30 (29.4%)	24 (23.5%)	1.354, (0.725–2.530)	0.342 ^a^
	GG	17 (16.6%)	16 (15.7%)	1.075, (0.510–2.266)	0.849 ^a^
***IL*-8 ** **(+781 *C*/*T*)**	CC	28 (27.5%)	21 (20.5%)	1.286, (0.676–2.445)	0.444 ^a^
	CT	27 (26.5%)	26 (25.5%)	1.052, (0.563–1.968)	0.873 ^a^
	TT	-	-	-	-
***Genotype Distributions***		***CAD Group*** **(*n* = 102), *Smoking* (+/−)**		***OR*, ** ** 95% *CI***	***p-Value***
***IL*-18 ** **(−607 *C*/*A*)**	CC	16 (15.7%)	12 (11.8%)	1.395, (0.624–3.120)	0.417 ^a^
	CA	35 (34.3%)	21 (20.5%)	2.015, (1.073–3.785)	**0.029 *^a^*** *
	AA	12 (11.8%)	6 (5.9%)	2.133, (0.768–5.924)	0.146 ^a^
***IL*-18 ** **(−137 *G*/*C*)**	CC	11 (10.8%)	5 (4.9%)	2.345, (0.784–7.011)	0.127 ^a^
	GC	33 (32.4%)	19 (18.6%)	2.089, (1.092–3.996)	**0.026 *^a^*** *
	GG	19 (18.6%)	15 (14.7%)	1.328, (0.633–2.785)	0.453 ^a^
***IL*-8 ** **(+781 *C*/*T*)**	CC	28 (27.5%)	20 (19.6%)	1.551, (0.806–2.984)	0.188 ^a^
	CT	35 (34.3%)	19 (18.6%)	2.282, (1.198–4.348)	**0.012 *^a^*** *
	TT	-	-	-	-
***Genotype Distributions***		***CAD Group*** **(*n* = 102), *Alcohol* (+/−)**		***OR*, ** ** 95% *CI***	***p-Value***
***IL*-18 ** **(−607 *C*/*A*)**	CC	11 (10.8%)	20 (19.6%)	0.496, (0.224–1.096)	0.083 ^a^
	CA	18 (17.6%)	38 (37.3%)	0.361, (0.189–0.690)	**0.002 *^a^*** *
	AA	5 (4.9%)	10 (9.8%)	0.474, (0.156–1.440)	0.188 ^a^
***IL*-18 ** **(−137 *G*/*C*)**	CC	12 (11.8%)	9 (8.8%)	1.378, (0.554–3.428)	0.491 ^a^
	GC	19 (18.6%)	33 (32.4%)	0.479, (0.250–0.916)	**0.026 *^a^*** *
	GG	3 (2.9%)	26 (25.5%)	0.089, (0.026–0.304)	**<0.001 *^a^*** *
***IL*-8 ** **(+781 *C*/*T*)**	CC	17 (16.6%)	31 (30.5%)	0.458, (0.234–0.895)	**0.022 *^a^*** *
	CT	17 (16.6%)	37 (36.3%)	0.351, (0.182–0.679)	**0.002 *^a^*** *
	TT	-	-	-	-
***Genotype Distributions***		***CAD Group*** **(*n* = 102), *Family History of CAD* (+/−)**		***OR*, ** ** 95% *CI***	***p-Value***
***IL*-18 ** **(−607 *C*/*A*)**	CC	22 (21.5%)	8 (7.9%)	3.231, (1.364–7.654)	**0.008 *^a^*** *
	CA	23 (22.6%)	34 (33.3%)	0.582, (0.313–1.083)	0.088 ^a^
	AA	12 (11.8%)	3 (2.9%)	4.400, (1.203–16.096)	**0.025 *^a^*** *
***IL*-18 ** **(−137 *G*/*C*)**	CC	26 (25.5%)	4 (3.9%)	8.382, (2.805–25.041)	**<0.001 *^a^*** *
	GC	20 (19.6%)	26 (25.5%)	0.713, (0.368–1.381)	0.316 ^a^
	GG	11 (10.8%)	15 (14.7%)	0.701, (0.305–1.611)	0.403 ^a^
***IL*-8 ** **(+781 *C*/*T*)**	CC	40 (39.3%)	17 (16.6%)	3.226, (1.675–6.211)	**<0.001 *^a^*** *
	CT	15 (14.7%)	25 (24.5%)	0.531, (0.261–1.080)	0.081 ^a^
	TT	2 (2.0%)	3 (2.9%)	1.515, (0.248–9.265)	0.653 ^a^

^a^ Logistic regression. OR: Odds ratio. CI: Confidence interval. CAD: Coronary artery disease. * Statistical significance (*p* < 0.05), given in bold italic font. Note: *p*-values in this table reflect nominal significance. No adjustment for multiple comparisons was performed. These results are exploratory and intended to guide future research.

**Table 3:** Logistic regression analysis and odds ratio values of gene variation genotype distributions.

*Genotype Distributions*		*CAD Group* (*n* = 102)	*Control Group* (*n* = 102)	*p-Value*
***IL*-18 ** **(−607 *C*/*A*)**	CC	30 (29.4%)	25 (24.5%)	***0.013 ^a^*** *
	CA	54 (53.0%)	71 (69.6%)	
	AA	18 (17.6%)	6 (5.9%)	
***IL*-18 ** **(−137 *G*/*C*)**	CC	18 (17.6%)	5 (4.9%)	0.012 ^a^*
	GC	51 (50.0%)	64 (62.7%)	
	GG	33 (32.4%)	33 (32.4%)	
***IL*-8 ** **(+781 *C*/*T*)**	CC	49 (48.0%)	28 (27.5%)	** <*0.001 ^a^*** *
	CT	51 (50.0%)	52 (51.0%)	
	TT	2 (2.0%)	22 (21.5%)	
***IL*-18 ** **(−607 *C*/*A*)**	CC	OR = 1.283 (0.690–2.387)		0.431 ^b^
	CA	OR = 0.491 (0.277–0.872)		***0.015 ^b^*** *
	AA	OR = 3.429 (1.301–9.038)		***0.013 ^b^*** *
***IL*-18 ** **(−137 *G*/*C*)**	CC	OR = 4.157 (1.480–11.679)		***0.007 ^b^*** *
	GC	OR = 0.594 (0.340–1.038)		0.067 ^b^*
	GG	OR = 1.000 (0.556–1.798)		1.000 ^b^*
***IL*-8 ** **(+781 *C*/*T*)**	CC	OR = 2.443 (1.364–4.378)		***0.003 ^b^*** *
	CT	OR = 0.962 (0.555–1.665)		0.889 ^b^*
	TT	OR = 0.073 (0.017–0.319)		** <*0.001 ^b^*** *

^a^ Chi-squared test. ^b^ Logistic regression. OR: odds ratio. CAD: coronary artery disease. * Statistical significance (*p* < 0.05), given in bold italic font. CC: Cytosine–cytosine; CA: Cytosine–adenine; CT: Cytosine–thymine; TT: Thymine–thymine; AA: Adenine–adenine; GG: Guanine–guanine; GC: Guanine–cytosine.

**Table 4:** Haplotype and multi-locus genotype combination analysis, frequencies, and odds ratio values for IL-18 and IL-8 gene variations.

*Haplotype and Combined Genotype Analysis* *IL-18 (−607 C/A)/IL-18 (−137 G/C)*	*Patients with CAD* (*N* = 102)	*Frequency* (%)	*Controls* (*N* = 102)	*Frequency* (%)	*OR,* 95% *CI, p-Value*
CC-CC	1	**1.0**	1	**1.0**	1.000, (0.062–16.209), *p* = 1.000
CA-CC	15	**14.7**	4	**3.9**	4.224, (1.351–13.209), *p* = 0.013 *
AA-CC	3	**2.9**	1	**1.0**	3.061, (0.313–29.926), *p* = 0.336
CC-GC	17	**16.6**	13	**12.7**	1.369, (0.627–2.990), *p* = 0.430
CA-GC	27	**26.5**	47	**46.1**	0.421, (0.234–0.758), *p* = 0.004 *
AA-GC	7	**6.9**	3	**2.9**	2.432, (0.611–9.680), *p* = 0.208
CC-GG	12	**11.8**	11	**10.8**	1.103, (0.463–2.629), *p* = 0.825
CA-GG	16	**15.7**	20	**19.6**	0.763, (0.370–1.573), *p* = 0.463
AA-GG	4	**3.9**	2	**2.0**	2.041, (0.365–11.398), *p* = 0.416
***IL*-18 ** ** (−607 *C*/*A*)/*IL*-8 (+781 *C*/*T*)**					
CC-CC	14	**13.7**	5	**4.9**	3.086, (1.068–8.918), *p* = 0.037 *
CA-CC	22	**21.5**	19	**18.6**	1.201, (0.605–2.386), *p* = 0.600
AA-CC	12	**11.8**	4	**3.9**	3.267, (1.017–10.496), *p* = 0.046 *
CC-CT	15	**14.7**	12	**11.8**	1.293, (0.573–2.919), *p* = 0.536
CA-CT	30	**29.4**	37	**36.3**	0.732, (0.407–1.316), *p* = 0.297
AA-CT	5	**4.9**	3	**2.9**	1.701, (0.396–7.314), *p* = 0.475
CC-TT	2	**2.0**	10	**9.8**	0.184, (0.039–0.862), *p* = 0.032 *
CA-TT	2	**2.0**	12	**11.8**	0.150, (0.033–0.689), *p* = 0.015 *
***IL*-18 ** ** (−137 *G*/*C*)/*IL*-8 (+781 *C*/*T*)**					
CC-CC	4	**3.9**	1	**1.0**	4.122, (0.453–37.538), *p* = 0.209
GC-CC	22	**21.5**	16	**15.7**	1.478, (0.725–3.014), *p* = 0.282
GG-CC	22	**21.5**	11	**10.8**	2.275, (1.039–4.981), *p* = 0.040 *
CC-CT	8	**7.9**	3	**2.9**	2.809, (0.723–10.906), *p* = 0.136
GC-CT	29	**28.4**	35	**34.3**	0.761, (0.420–1.377), *p* = 0.366
GG-CT	13	**12.8**	17	**16.6**	0.730, (0.335–1.595), *p* = 0.430
CC-TT	1	**1.0**	1	**1.0**	1.000, (0.062–16.209), *p* = 1.000
GC-TT	2	**2.0**	12	**11.8**	0.150, (0.033–0.689), *p* = 0.015 *
GG-TT	1	**1.0**	6	**5.9**	0.158, (0.019–1.340), *p* = 0.091

OR: Odds ratio. CI: Confidence interval. CAD: Coronary artery disease. * Statistical significance (*p* < 0.05), given in bold italic font.

## Data Availability

The data are not publicly available due to ethical reasons.

## Supplementary Materials

The following supporting information can be downloaded at: https://www.mdpi.com/article/10.3390/cimb48060589/s1.

## Abbreviations

CAD	Coronary artery disease
IL-18	Interleukin 18
IL-8	Interleukin 8
EDTA	Ethylenediaminetetraacetic acid
PCR	Polymerase chain reaction
RFLP	Restriction fragment length polymorphism
