# Supplementary material for: Inflammatory Cytokine Genetics and Coronary Artery Disease: Pathogenetic and Protective Analysis of IL-18 (−607 C/A, −137 G/C) and IL-8 (+781 C/T) Gene Variations
Source: Curr Issues Mol Biol. 2026 Jun 2;48(6):589. doi: 10.3390/cimb48060589 (PMC13298403; doi:10.3390/cimb48060589)
Supplement: Supplementary file 1 [file cimb-48-00589-s001.zip › Supplemental File S1.docx]

**Supplemental File S1.** Methodological Details for Allele-Specific PCR

| ***Gene Variation*** | ***Primer Sequences (5' - 3')*** | ***PCR Conditions*** | ***Expected Product Lengths*** |
| --- | --- | --- | --- |
| ***IL-18***  ***(-607 C/A)*** | **RP:** 5ʹ-TAACCTCATTCAGGACTTCC-3ʹ  **F1P:** 5ʹ-GTTGCAGAAAGTGTAAAAATTATTAC-3ʹ  **F2P:** 5ʹ-GTTGCAGAAAGTGTAAAAATTATTAA-3ʹ  **FCP:** 5ʹ-CTTTGCTATCATTCCAGGAA-3ʹ | 3 minutes at 94^0^C  20 seconds at 94^0^C  20 seconds at 50^0^C 40 cycle  20 seconds at 72^0^C  5 minutes at 72^0^C | **CC** 196bp (F1P)  **CA** 196bp  (F1P and F2P)  **AA** 196bp (F2P) |
| ***IL-18***  ***(-137 G/C)*** | **RP:** 5ʹ-AGGAGGGCAAAATGCACTGG-3ʹ  **F1P:** 5ʹ-CCCCAACTTTTACGGAAGAAAAG-3ʹ  **F2P:** 5ʹ-CCCCAACTTTTACGGAAGAAAAC-3ʹ  **FCP:** 5ʹ-CCAATAGGACTGATTATTCCGCA-3ʹ | 3 minutes at 94^0^C  20 seconds at 94^0^C  20 seconds at 54^0^C 40 cycle  20 seconds at 72^0^C  5 minutes at 72^0^C | **GG** 261bp (F1P)  **GC** 261bp  (F1P and F2P)  **CC** 261bp (F2P) |

This table describes the parameters for the IL-18 promoter variations, which do not require restriction enzymes.

Allele-Specific PCR Components: 50 ng DNA, forward/reverse primers, 1x PCR buffer, 3mM MgCl_2_, 1.25 U Taq DNA polymerase.

**Visualization:** PCR products were observed in 2% agarose gel electrophoresis.

**RP:** Reverse primer; **F1P:** Forward 1 primer; **F2P:** Forward 2 primer; **FCP:** Forward control primer.
