# Supplementary material for: Inflammatory Cytokine Genetics and Coronary Artery Disease: Pathogenetic and Protective Analysis of IL-18 (−607 C/A, −137 G/C) and IL-8 (+781 C/T) Gene Variations
Source: Curr Issues Mol Biol. 2026 Jun 2;48(6):589. doi: 10.3390/cimb48060589 (PMC13298403; doi:10.3390/cimb48060589)
Supplement: Supplementary file 1 [file cimb-48-00589-s001.zip › Supplemental File S2.docx]

**Supplemental File S2.** Methodological Details for PCR-RFLP

| ***Gene Variation*** | ***Primer Sequences (5' - 3')*** | ***PCR Conditions*** | ***Restriction Enzyme*** | ***Digested Product Lengths*** |
| --- | --- | --- | --- | --- |
| ***IL-8***  ***(+781 C/T)*** | **FP:** 5’-CTCTAACTCTTTATATAGGAATT-3’  **RP:** 5’-GATTGATTTTATCAACAGGCA-3’ | 5 minutes at 94^0^C  1 minute at 94^0^C  1 minute at 61^0^C 35 cycle  2 minutes at 72^0^C  20 minutes at 72^0^C | EcoRI | **TT** 203bp (Undigested)  **CT** 203bp, 184bp, 19bp  **CC** 184bp, 19bp  (19bp is not observed) |

This table describes the parameters for the IL-8 variation, involving post-PCR enzymatic digestion.

**RFLP Components:** PCR reaction products, 1x Buffer Tango, dH_2_O, 5 Units of EcoRI restriction enzyme.

**Visualization:** RFLP products were observed in 2.5% agarose gel electrophoresis

(Note: 19bp fragment is not typically observed).

**FP:** Forward primer; **RP:** Reverse primer
