# Supplementary material for: Inflammatory Cytokine Genetics and Coronary Artery Disease: Pathogenetic and Protective Analysis of IL-18 (−607 C/A, −137 G/C) and IL-8 (+781 C/T) Gene Variations
Source: Curr Issues Mol Biol. 2026 Jun 2;48(6):589. doi: 10.3390/cimb48060589 (PMC13298403; doi:10.3390/cimb48060589)
Supplement: Supplementary file 1 [file cimb-48-00589-s001.zip › Supplemental File S3.docx]

**Supplemental File S3.** Comparison of clinical and demographic parameters between patient with coroner arter disease and healthy control groups

| ***Clinical and demographic parameters*** | ***Patient***  ***group (n=102)*** | ***Control***  ***group (n=102)*** | ***OR; 95% Confidence Interval*** | ***p*** |
| --- | --- | --- | --- | --- |
| Age | 66.918 ± 10.676 | 66.357 ± 7.899 | 0.561; 2.058-3.180 | 0.673^a^ |
| Hypertension (+) | 58 (56.9%) | 15 (14.7%) | 7.646; 3.898-14.996 | ***<0.001^b*^*** |
| Diabetes Mellitus (+) | 60 (58.8%) | 17 (16.7%) | 7.143; 3.717-13.726 | ***<0.001^b*^*** |
| Cholesterol (+) | 55 (53.9%) | 13 (12.7%) | 8.012; 3.978-16.136 | ***<0.001^b*^*** |
| Familial history  of CAD (+) | 57 (55.9%) | 17 (16.7%) | 6.333; 3.303-12.145 | ***<0.001^b*^*** |
| Alcohol (+) | 34 (33.3%) | 22 (21.6%) | 1.818; 0.972-3.401 | 0.061^b^ |
| Smoking (+) | 63 (61.8%) | 31 (30.4%) | 3.700; 2.070-6.614 | ***<0.001^b*^*** |

^a^Independent Samples Test

^b^Logistic Regression

(+): Available; *: Significance (p<0,05)

**CAD:** Coroner Arter Disease
