# Supplementary figures and images for: Inflammatory Cytokine Genetics and Coronary Artery Disease: Pathogenetic and Protective Analysis of IL-18 (−607 C/A, −137 G/C) and IL-8 (+781 C/T) Gene Variations
Source: Curr Issues Mol Biol. 2026 Jun 2;48(6):589. doi: 10.3390/cimb48060589 (PMC13298403; doi:10.3390/cimb48060589)

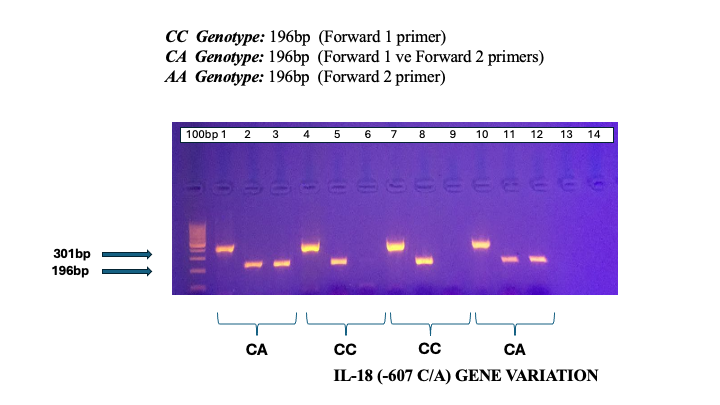

Supplement: Supplementary file 1 [file cimb-48-00589-s001.zip › Figure S1.tiff]

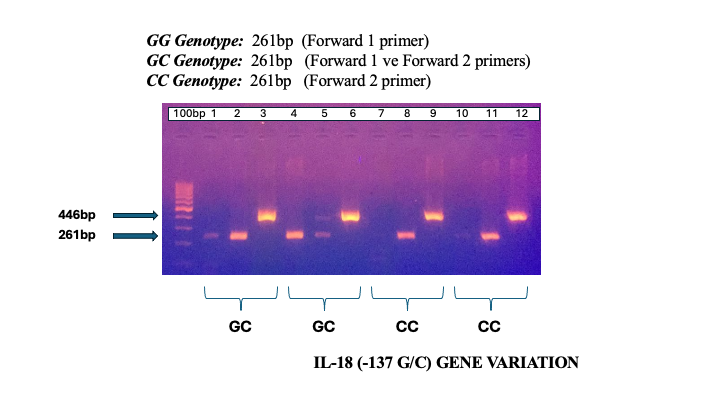

Supplement: Supplementary file 1 [file cimb-48-00589-s001.zip › Figure S2.tiff]

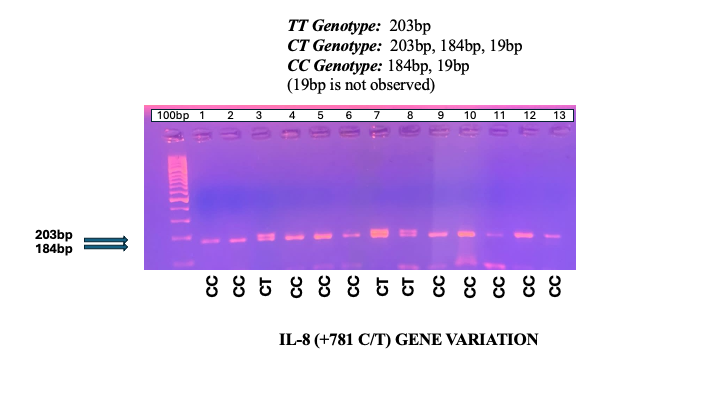

Supplement: Supplementary file 1 [file cimb-48-00589-s001.zip › Figure S3.tiff]
